# Snake bite - cytotoxic effects of snake venom: a rare clinical image

**DOI:** 10.11604/pamj.2023.44.61.37759

**Published:** 2023-02-01

**Authors:** Lalrintluangi Royte, Achita Sawarkar

**Affiliations:** 1 Department of Community Health Nursing, Smt. Radhikabai Meghe Memorial College of Nursing, Datta Meghe Institute of Medical Sciences, Sawangi (Meghe), Wardha, Maharashtra, India

**Keywords:** Cytotoxic, snake bite, snake venom, envenomation, tissue necrosis

## Image in medicine

Venoms of cobras contain high abundances of cytotoxins, which contribute to tissue necrosis in cobra envenomation. Cyto refers to cells, and cytotoxicity broadly describes a toxic effect on cell function. Cytotoxic activity can lead to edema (fluid retention), severe blistering, apoptosis (cell death), and necrosis. As the name suggests, cytotoxic venom kills cells. This venom is not as deadly as hemotoxic or neurotoxic venom. However, secondary injuries such as loss of limb function and other disabilities often result from cytotoxic venom. A 38-year-old male was brought to out-patient department with a complaint of necrotic tissue on the right side of the dorsal hand and wrist which results from an untreated snake bite roughly for about one week. Physical examination was performed by the physician which shows severe local tissue damage on the wrist and dorsal part of the right hand. Necessary treatments were given and the patient was referred to the medicine department for further management.

**Figure 1:**
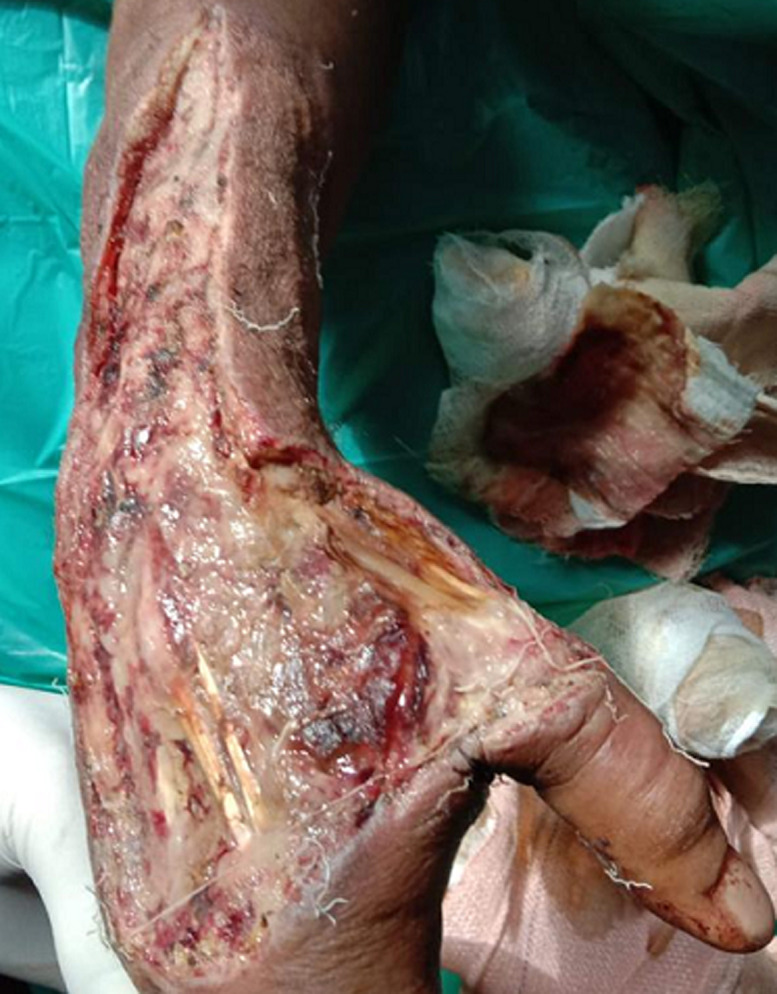
damaged tissue on the dorsal hand

